# NMDA Receptor Modulators in the Treatment of Drug Addiction

**DOI:** 10.3390/ph6020251

**Published:** 2013-02-06

**Authors:** Seven E. Tomek, Amber L. LaCrosse, Natali E. Nemirovsky, M. Foster Olive

**Affiliations:** 1 Department of Psychology, Arizona State University, Tempe, AZ 85287, USA; E-Mails: seven.tomek@asu.edu (S.E.T.), alacross@asu.edu (A.L.L.), nnemirov@asu.edu (N.E.N.); 2 Interdisciplinary Graduate Program in Neuroscience, Arizona State University, Tempe, AZ 85287, USA; E-Mail: foster.olive@asu.edu

**Keywords:** glutamate, *N*-methyl-d-aspartate receptor, glycine binding site, antagonist, partial agonist

## Abstract

Glutamate plays a pivotal role in drug addiction, and the *N*-methyl-d-aspartate (NMDA) glutamate receptor subtype serves as a molecular target for several drugs of abuse. In this review, we will provide an overview of NMDA receptor structure and function, followed by a review of the mechanism of action, clinical efficacy, and side effect profile of NMDA receptor ligands that are currently in use or being explored for the treatment of drug addiction. These ligands include the NMDA receptor modulators memantine and acamprosate, as well as the partial NMDA agonist d-Cycloserine. Data collected to date suggest that direct NMDA receptor modulators have relatively limited efficacy in the treatment of drug addiction, and that partial agonism of NMDA receptors may have some efficacy with regards to extinction learning during cue exposure therapy. However, the lack of consistency in results to date clearly indicates that additional studies are needed, as are studies examining novel ligands with indirect mechanisms for altering NMDA receptor function.

## 1. Introduction

Substance abuse and dependence are cardinal issues of public health that do not discriminate between race, ethnicity, gender, or socioeconomic status of drug users. Recent estimates of licit and illicit substance use and abuse revealed that a concerning number of individuals are directly affected by substance use disorders (SUDs), with more than tens of millions of people reported to have drug-related problems worldwide [1]. According to the 2009 National Survey on Drug Use and Health (NSDUH), more than 20 million Americans over the age of 12 meet the criteria for a SUD [2]. Some of the most commonly abused substances include alcohol, nicotine, marijuana, amphetamines, cocaine, heroin, and prescription medications. Both licit and illicit drugs are used for a variety of reasons, including altering mental state, experience of rewarding effects, performance enhancement, and self-medication. In subsets of individuals, chronic drug use results in dependency that manifests as an overpowering desire for the drug and impairment in controlling drug intake and drug-seeking behavior [3]. Although there have been some advances in behavioral and pharmacological approaches to the treatment of SUDs, these disorders continue to maintain their presence in society, illustrating the necessity for further research on the underlying neuropathological events that predispose to precipitate SUDs.

Despite the notion that substance abuse often leads to substance dependence, substance abuse and dependence are in fact separate disorders with distinct criteria characteristics as defined in the 4th edition of the American Psychiatric Association’s Diagnostic and Statistical Manual of Mental Disorders (DSM-IV). Substance abuse is most commonly described as intentional misuse of a substance, which can include recurring maladaptive patterns of substance use despite having persistent or recurrent problems caused by or exacerbated by the effects of the substance. Drug use can result in physical, psychological, interpersonal, or legal problems. Substance dependence incorporates the aforementioned characteristics of misuse but also comprises signs of tolerance, withdrawal symptoms following cessation of drug use, increased quantity and/or frequency of use, as well as recurring but unsuccessful desire to stop or limit drug use [4]. Occasional or limited use of a substance with high potential for abuse is clinically distinct from substance dependence due to the behavioral and psychological characteristics of dependence, including escalated use of drug, inability to control limiting drug intake, and the development of chronic compulsive drug-seeking behavior. The distinction between substance use, abuse, and dependence is also reflected in observations that approximately 15.6% (29 million) of the U.S. adult population will participate in nonmedical or illicit drug use at some point in their lives, yet only 2.9% of the population will progress from use/abuse to substance dependence [5,6,7]. Substance dependence, frequently referred to as drug addiction, occurs through physiological changes that take place in the brain over the course of chronic drug use, resulting in cellular and molecular changes that lead to maladaptive behavioral patterns [8]. The distinction between substance abuse and dependence is fundamental for providing appropriate treatment due to the differences in acute as well as lasting neurobiological changes that each disorder engenders [for thorough review of neurocircuitry of addiction, refer to [5]).

In recent years, preclinical and clinical research has shown that there is considerable overlap between the neural substrates that normally serve reward-related learning in substance dependence and in non-drug “behavioral” addictions, including pathological gambling and kleptomania [9,10]. A recent review addresses all addictions as a “runaway phenomenon” that has directly affected almost half of the U.S. population, and includes “process” addictions such as eating, shopping, sex, internet, and exercise addiction [11,12]. From a historical standpoint, addiction has traditionally been viewed as solely pertaining to pharmacological substances. However, over the last several decades, substantial research investigating addictive behaviors has led to the notion that “behavioral” or “process” addictions might be better viewed as a separate category of disorders in future revisions of DSM-IV, which currently includes no diagnostic category for these disorders [12].

Although there have been a number of medications approved for other medical conditions that have been investigated as possible treatment aids for SUDs, in the U.S. there are only a handful of medications approved for treating specific addictions to substances such as nicotine, opiates, and alcohol. Standard pharmacological treatments for alcohol dependence include the aldehyde dehydrogenase inhibitor disulfiram, the broad spectrum opiate antagonist naltrexone, and the NMDA receptor modulator acamprosate. Standard pharmacological treatments for opiate dependence are generally opioid substitution therapies such as methadone and buprenorphine, the latter of which is often formulated with low doses of the opiate antagonist naloxone to deter abuse. Pharmacological treatments for nicotine dependence include nicotine replacement therapies, the monoamine uptake inhibitor buproprion, and the partial nicotinic acetylcholine receptor agonist varenicline. To date, there are no approved medications specifically for the treatment of addiction to cocaine, methamphetamine, or marijuana, nor are there any approved to treat behavioral addictions. Currently, many of the medications developed for the treatment of SUDs have shown very modest efficacy, likely due to poor medication compliance and adverse side effects [9].

In the past, much attention has been given to the neurobiological substrates that underlie the rewarding and reinforcing effects of drugs of abuse, focusing primarily on the mesolimbic dopamine reward circuitry. In the last several decades, however, it has become apparent that glutamatergic transmission plays a pivotal role in addiction and thus may be a key target for possible novel pharmacological treatments [13,14]. Glutamate, or L-glutamic acid, is the main excitatory neurotransmitter in the central nervous system (CNS) and can bind three different classes of ionotropic glutamate receptors (iGluRs) and three different classes of metabotropic glutamate receptors (mGluRs), each of which with its own distinct distribution in the nervous system, pharmacology, and signaling mechanisms. Glutamate synthesis, metabolism, receptor trafficking, signaling, and excitatory transmission are crucial components for normal brain functioning [15]. With regard to the mesolimbic dopamine reward circuitry, there are numerous glutamatergic innervations from distinct cell populations to the nucleus accumbens (NAcc), amygdaloid complex (Amyg), ventral tegmental area (VTA), and frontal cortex (FC) (for a review see [13]). The *N*-methyl-d-aspartate (NMDA) receptor is one of three types of iGluRs, and is critically involved in numerous neuronal and brain functions including fast excitatory transmission, synaptic plasticity, learning, and memory [16,17,18,19].

**Figure 1:**
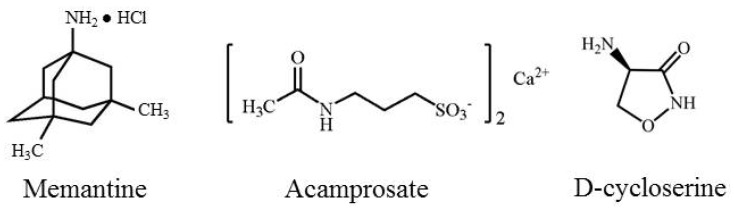
Chemical structures of memantine, acamprosate, and d-Cycloserine.

The following sections of this review will summarize NMDA receptor structure and function, followed by a review of the mechanism of action, clinical efficacy, and adverse side effects of NMDA receptor ligands that are currently in use or under investigation for the treatment of SUDs. These compounds include the NMDA receptor modulators memantine and acamprosate, and the partial NMDA agonist d-Cycloserine (see Figure 1).

## 2. NMDA Receptor Structure, Expression Patterns, and Pharmacology

Three general classes of iGluRs include the NMDA, 2-amino-3-(3-hydroxy-5-methylisoxazol-4-yl)propanoic acid (AMPA), and kainic acid (KA) receptors. The NMDA receptor has long been known to influence synaptic plasticity and long-term potentiation (LTP), both of which alter physical elements within the synapse to increase the functioning and efficiency of neurotransmission. Synaptic plasticity and LTP are critical for processes related to learning and memory [18,20]. Abnormal functioning of the NMDA receptor is theorized to be associated with several diseases, including schizophrenia, epilepsy, Alzheimer's disease, motor dysfunction, and drug addiction. Abnormal functioning of the receptor can include hyper- or hypo-activation by glutamate, endogenous neuromodulators, as well as exogenous pharmacological ligands. Hyperactivation of the NMDA receptor results in an excessive influx of Ca^2+^, which causes excitotoxicity [21,22] and ultimately leads to cell death and possibly disease progression. Hypoactivation of the NMDA receptor has been linked to hallucinations, coma, and developmental abnormalities [21,23,24]. Because of the high sensitivity of the NMDA receptor to modulation and the propensity towards adverse side effects and neurotoxicity, effective therapeutic manipulation has proved to be rather difficult.

Group I mGluRs (mGluR1 and mGluR5) are physically and biochemically linked to the NMDA receptors through their intracellular signaling pathways and scaffolding proteins such as postsynaptic density 95 (PSD-95) and synaptic associated protein 102 (SAP-102) [20]. Activation of Group I mGluRs can facilitate NMDA receptor activity, thus offering an indirect mechanism for enhanced NMDA receptor function. Negative allosteric modulators (NAMs) of Group I mGluRs have the opposite effect [25]. Group II (mGluR2 and mGluR3) and Group III (mGluR4, mGluR6, mGluR7, and mGluR8) are negatively coupled to adenylyl cyclase, but show little indirect modulation of NMDA receptor function.

**Figure 2:**
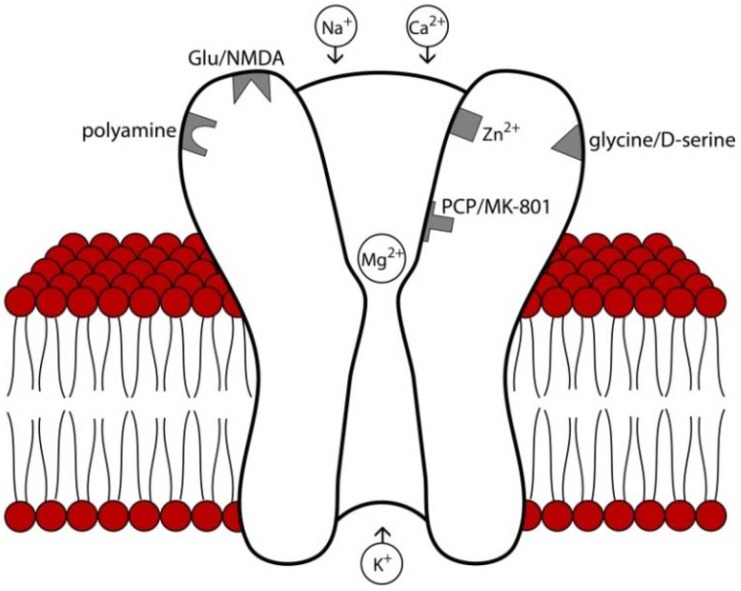
Structure and major binding sites of the NMDA receptor. At rest, the receptor pore is blocked by Mg^2+^ which must be removed by slight membrane to allow cation conductance. Binding sites for glutamate, the endogenous co-agonists D-serine and glycine, and endogenous modulators such as polyamines, Zn^2+^, and protons are primarily localized to extracellular domains. Psychomimetic NMDA antagonists such as phencyclidine (PCP) and MK-801 bind to deep regions of the channel pore. Relevant to the pharmacological agents reviewed here, memantine blocks superficial regions of the channel pore [31], acamprosate is believed to interact with the polyamine binding site [32], and binding d-Cycloserine binds to the D-serine/glycine co-agonist site [33].

NMDA receptors are heterotetrameric cation channels comprised of a ubiquitous NR1 subunit and three others from the family of NR2 and NR3 subunits. The receptor itself has an extracellular *N*-terminus and can be manipulated by protons or polyamines aside from its orthogonal binding site. Each subunit has four transmembrane domains (M1-M4), and a cytoplasmic *C*-terminal domain is present intracellularly and interacts with intracellular signaling proteins [20,26]. The NMDA receptor is permeable to both Na^+ ^and Ca^2+ ^[26], but during the resting state the channel is blocked by Mg^2+ ^[20], which requires slight membrane depolarization for removal and cationic conductance (see Figure 2). Endogenous binding sites on the NMDA receptor include glutamate, the endogenous co-agonists glycine and D-serine, Zn^2+^, H^+^, and polyamines. Currently there are eight different splice variants known for the NR1 subunit [27], and NR2 and NR3 subunits are each encoded by families of different genes [28]. Following channel opening and cationic conductance, several intracellular signaling pathways activated including protein kinase A and protein kinase, which can in turn activate pathways that regulate expression and trafficking of NMDA as well as other iGluR and mGluR receptors [20]. NMDA receptor subunits have specific neuroanatomical expression patterns [17]. Although the distribution patterns in the human brain have been less extensively explored, they appear to be similar but not identical to that of rodents [29], with high levels of expression in regions important for memory and higher order cognition such as the hippocampus and cerebral cortex [30].

Addiction is thought to arise from cellular and molecular changes in the brain produces by chronic use [34], including changes in neurotransmission, dendritic structure, gene expression, epigenetic chromatin modifications, and synaptic plasticity [8]. Much of the early evidence for a role of the NMDA receptor in addictive processes came from behavioral pharmacology studies in rodents using the conditioned place preference or intravenous self-administration paradigms. These studies demonstrated that NMDA antagonists block the rewarding or reinforcing effects of drugs of abuse such as morphine and cocaine [35,36]. As we now will review, there is ample evidence that the NMDA receptor is involved in addiction in humans but is also a potential yet elusive therapeutic target.

## 3. NMDA Receptor Modulators

### 3.1. Memantine

#### 3.1.1. Mechanism of Action

Memantine is approved for treatment of cognitive decline in moderate to severe Alzheimer's disease [37]. This compound is derived from amantadine and blocks the NMDA receptor channel much like Mg^2+^ [38]. However, unlike Mg^2+^, it blocks the NMDA channel with a higher affinity and less voltage dependency. In addition, recent evidence suggests that memantine preferentially occupies a more superficial region of the channel pore than NMDA receptors with more psychotomimetic effects such as ketamine and PCP (see Figure 1) [31]. Memantine is considered an “uncompetitive antagonist” since it binds to the receptor channel rather than the extracellular glutamate binding site [38]. In addition to its antagonist actions at NMDA receptors, memantine also blocks the type 3 serotonin (5-HT_3_) receptor as well as nicotinic acetylcholine receptors [9]. Memantine has been shown to block NMDAR activity in the presence of prolonged elevations of glutamate concentrations, but it is not as active when glutamate levels increase for shorter periods of time, as in synaptic transmission [26]. Some studies have suggested that memantine preferentially blocks extrasynaptic NMDAR channels while sparing normal synaptic activity, which may underlie the general tolerability of memantine. Unlike other NMDA antagonists such as ketamine or dextromethorphan, memantine does not appear to have abuse potential [9].

#### 3.1.2. Preclinical Findings

Studies in mice and rats have shown that memantine attenuates the acquisition or maintenance of intravenous self-administration of morphine, nicotine, or cocaine [39,40,41] as well as the conditioned rewarding effects of these drugs [42,43,44,45,46,47,48,49], suggesting a possible therapeutic role for this compound in opiate, nicotine, and cocaine dependence. However, it was also shown that memantine failed to suppress the reinstatement of cocaine-seeking behavior [50], an established model of relapse. However, to our knowledge no clinical trials on memantine for addiction to these substances have been published.

#### 3.1.3. Clinical Efficacy

Two studies have shown that patients taking memantine reported decreased craving of alcohol [51,52], one study (n = 38) showed decreased symptoms of alcohol withdrawal [52], two studies (n = 20 and n = 34) demonstrated decreased quantity of alcohol consumed [53,54]. However, other studies have shown a lack of effect of memantine on on-going alcohol consumption [37]. In studies of methamphetamine dependence, it has been demonstrated that memantine-methamphetamine combinations produce novel discriminative stimulus effects, and that memantine alone can produce some stimulant-like subjective effects (n = 6) [55]. Thus, the overall efficacy of memantine for the treatment of alcohol use disorders appears minimal at best, and there is no compelling basis for its use in methamphetamine addiction.

#### 3.1.4. Adverse Side Effects

Memantine is generally well tolerated at therapeutic doses. Severe drug interactions with memantine are rare, but moderate interactions of memantine with bupropion have been reported, as memantine may increase the plasma concentrations of bupropion and cause bupropion toxicity which can manifest as agitation, anxiety, tremors, insomnia, and seizures. Concomitant use of memantine and anti-Parkinsonian drug trihexyphenidyl may increase the anticholinergic effects of trihexyphenidyl, which include dry mouth, blurry vision, or urinary issues.

### 3.2. Acamprosate

#### 3.2.1. Mechanism of Action

Acamprosate is prescribed to help people dependent on alcohol maintain abstinence with the support of counseling. Acamprosate is a synthetic compound derived from homotaurine, a nonspecific GABA agonist. It is structurally similar to amino acids such as taurine, glutamate, and GABA, and is formulated as calcium salt to aid in its absorption from the gastrointestinal tract (Figure 1). Acamprosate is also N-acetylated to facilitate crossing of the blood brain barrier [56].

The neuropharmacological mechanisms underlying the actions of acamprosate have been difficult to elucidate. Originally, acamprosate was thought to exert its effects via a GABAergic mechanism, since the drug has a chemical structure similar to that of GABA [57]. However, other studies have failed to find any direct evidence of acamprosate binding to or facilitation of GABA_A_ receptor function [58,59]. Despite this, facilitation of GABAergic transmission by acamprosate may occur via blockade of inhibitory presynaptic GABA_B_ autoreceptors [59]. Zeise and colleagues [60,61] were the first demonstrate antagonistic activity of acamprosate at NMDA receptors, which has been confirmed by others [62,63]. Yet some investigators have found opposite effects, with acamprosate actually potentiating NMDA receptor [58,59], while still others found no effect of acamprosate on NMDA-mediated synaptic transmission [64]. These inconsistencies are perhaps related to factors such as brain region examined, NMDA receptor subunit composition, state of neuronal excitation, and the presence of various endogenous NMDA receptor neuromodulators such as polyamines [56,65]. Binding studies have suggested an interaction of acamprosate with the spermidine-, glutamate- and/or MK-801-sensitive binding site of the NMDA receptor [32,66]. On the whole, there is a general consensus that acamprosate is an NMDA receptor modulator that restore the imbalance between excitatory and inhibitory neurotransmission caused by chronic alcohol exposure [56], likely at the polyamine site on the NMDA receptor complex [56]. Acamprosate may have differential effects on NMDA receptors at low concentrations, and on GABA_A_ receptors at higher concentrations [56].

#### 3.2.2. Preclinical Findings

Only one study in animals showing that acamprosate reduced voluntary ethanol consumption in rats [57] was published prior to the first demonstration of its clinical efficacy in reducing the incidence of relapse in alcoholics [67]. Other studies have showed similar reductions in alcohol intake by acamprosate in laboratory rodents (reviewed in [56,68]). As for drugs of abuse other than alcohol, there have been reports that acamprosate can reduce the acquisition or reinstatement of cocaine reward [69,70] as well as the reinstatement of cocaine-seeking following intravenous self-administration [71]. However, other studies have shown a lack of effect of acamprosate on heroin self-administration [72]

#### 3.2.3. Clinical Efficacy

The clinical efficacy of acamprosate has been studied throughout the world [73], however the results have been conflicting. Studies looking at overall alcohol consumption, subjective measures of alcohol craving, and promoting abstinence demonstrate effect sizes ranging from small to moderate [73,74]. However, there are also large multi-center studies such as the COMBINE study (n = 1,383) reporting that acamprosate is no more effective than the placebo in reducing alcohol related cravings or overall abstinence [75]. These discrepancies are still being investigated and debated, and it is likely that acamprosate is beneficial for the treatment of alcoholism in subsets of patients under certain treatment paradigms, settings, and desired outcome [56,76]. Following up on the aforementioned rodent studies indicating a possible role for acamprosate in treating cocaine dependence, one clinical trial (n = 60) examined the efficacy of acamprosate in reducing cocaine use in dependent individuals, but unfortunately the results of this trial were also negative [77]. Taken together, it appears that acamprosate has limited use in the treatment of SUDs.

#### 3.2.4. Adverse Side Effects

Acamprosate exerts very few adverse side effects. The most commonly reported side effect is diarrhea, likely due to the poor intestinal absorption of the drug [73]. Interactions of acamprosate with alcohol, diazepam, disulfiram, and naltrexone have not been reported.

### 3.3. d-Cycloserine (DCS)

#### 3.3.1. Mechanism of Action

d-Cycloserine (DCS, d-4-amino-3-isoxazolidone), a derivative of the naturally occurring amino acid d-serine, is an NMDA receptor partial agonist. It is generally prescribed to treat tuberculosis when other medications have shown to be ineffective, and is also used to treat certain urinary tract infections. It acts as co-agonist at the strychnine-insensitive glycine binding site on the NR1 subunit of the NMDA receptor. DCS increases the activation probability of the NMDA receptor; however, it requires the presence of glutamate binding to the receptor in order to exert its effects [78]. DCS activation enhances NMDA functioning by increasing calcium influx through these receptors without causing neurotoxicity [9,79]. However, DCS is less efficient than the endogenous ligands glycine and D-serine at modulating NMDA receptor function. High doses of DCS displace more efficacious endogenous ligands, and moderate doses of DCS have shown to facilitate NMDA receptor-dependent forms of synaptic plasticity and learning [78]. 

#### 3.3.2. Preclinical Findings

Potentiation of NMDA receptor function by DCS is believed to contribute to its ability to facilitate synaptic plasticity and certain forms of learning, including Pavlovian associative learning and extinction learning, and as such it has been reported to successfully facilitate the extinction of fear responses in anxiety disorder patients during cue exposure therapy in numerous clinical studies [16,80]. This area of research has recently been extended to the study of the extinction of the motivational salience of drug-related cues [78]. Rodent studies have shown DCS facilitates the extinction of a cocaine-induced conditioned place preference [81,82] as well as cocaine self-administration [83]. DCS also reduces reacquisition of cocaine self-administration by enhancing extinction learning [84] and when administered into the NAcc, attenuates the reinstatement of cocaine-seeking in a context-independent manner [85]. However, the timing of DCS administration may be highly important for the desired outcomes, since infusions of DCS into the basolateral amygdala following reactivation of cocaine-associated memories can actually potentiate the reconsolidation of these memories in cocaine self-administering rats [86]. As described below, these seemingly contradictory effects are also reflected in clinical literature.

#### 3.3.3. Clinical Efficacy

As enhancer of NMDA receptor function and thus a facilitator of certain forms of synaptic plasticity and learning, DCS is used in combination with cue exposure therapy (CET) to facilitate extinction of conditioned fear responses in various anxiety disorders including phobias and post-traumatic stress disorder [16,80,87]. SUDs, like anxiety disorders, involve conditioned responses to external and internal stimuli (cues). Cues that are associated with drug use and acute withdrawal elicit conditioned craving and withdrawal responses that contribute to recurring drug use and relapse [88,89]. Although CET alone has not been particularly effective in reducing drug-related conditioned responses in addicts, facilitation the extinction of these conditioning responses with DCS in theory could aid in improving the efficacy of this approach.

In a 2009 clinical study of nicotine-dependent cigarette smokers undergoing CET, Santa Ana *et al.* [90] found that administration of DCS significantly decreased physiological as well as subjective “urge to smoke” rating responses compared to placebo treatment (n = 26). Although there were no effects found on general smoking behavior during a follow up assessment, these preliminary findings supported the notion that DCS may be beneficial in combination with CET to augment effects of cues and adverse effects during attempts to quit smoking cigarettes [9,78]. On the contrary, a 2012 study by Kamboj *et al.* [91] involving n=32 subjects, which expanded on the Santa Ana *et al.* study [90] to include two CET sessions with DCS or placebo administration along with follow up assessments of smoking behavior, found no significant effects of DCS on cigarette cravings and smoking behavior (n=10). With these contradictory findings, it is of value to continue exploring possible alternative combinations of DCS and CET for nicotine addiction, paying particular attention to the timing of DCS administration to avoid promoting reconsolidation of drug cue reactivity.

Preliminary clinical findings by Price and colleagues [92,93] demonstrated rather unexpected findings from administration of DCS along with CET in cocaine-dependent patients. Unlike preclinical studies that have shown DCS to reduce reacquisition and enhanced extinction learning in animal models of cocaine addiction [82,83,84], these clinical studies demonstrated an *increase* in subjective reporting of cocaine craving in the DCS treatment group during the first of two CET sessions, and no statistically significant differences between placebo and DCS treatment in the second CET session nor follow up sessions [92]. Due to the small number of patients assessed (n = 10 and n = 32), as well compelling findings from preclinical studies on enhancement of drug-seeking by DCS, further investigations with different cue exposure paradigms and timing of DCS administration are clearly warranted.

Clinical studies on DCS treatment for alcohol-dependent subjects have found that DCS is either ineffective in reducing cue-elicited alcohol craving [94] or to produce a paradoxical increase in subjective reports of craving [95]. Similar lack of efficacy of DCS on the extinction of alcohol-seeking behavior in rodents have been reported [96].

Thus, although DCS appears to have theoretical promise as an addition to CET treatment for SUDs, disappointing clinical evidence suggests that more research should be conducted with variations in treatment plans, dose, and timing of administration of this pharmacotherapy to better explore clinical efficacy of DCS. A recent correspondence regarding clinical relevance of DCS and CET research for SU treatment suggests that current data may not be statistically significant due to both type I and type II errors [78,97]. There is also debate over the clinically utilized criteria in relapse prevention treatment, specifically regarding the clinical efficacy for treating SUDs when compared to the treatment of anxiety and fear disorders [97].

#### 3.3.4. Adverse Side Effects

Due to the limited amount of clinical data on DCS in the context of drug addiction, adverse effects are sparsely mentioned. However, some of the more common adverse effects of DCS in the general literature mainly include CNS manifestations such as headache, irritability, depression, psychosis, and convulsions. Drug interactions with DCS include alcohol and the antibiotic ethionamide. Alcohol is incompatible with DCS and can increase the risk of epileptic episodes, whereas ethionamide may cause neurotoxic side effects when used in combination with DCS.

## 4. Conclusions

With regards to the medications reviewed here that possess a glutamatergic mechanism of action (acamprosate, memantine, and d-Cycloserine), we conclude that neither acamprosate nor memantine show great promise as pharmacological adjuncts to psychosocial and behavioral interventioonds for SUDs. In addition, DCS has shown great promise in preclinical studies on extinction of drug-seeking as well as in human studies on the extinction of conditioned fear responses. However, evidence thus far on DCS as a successful treatment for SUDs has produced surprisingly contradictory results, with several clinical trials showing the DCS actually increasing drug craving.

**Figure 3:**
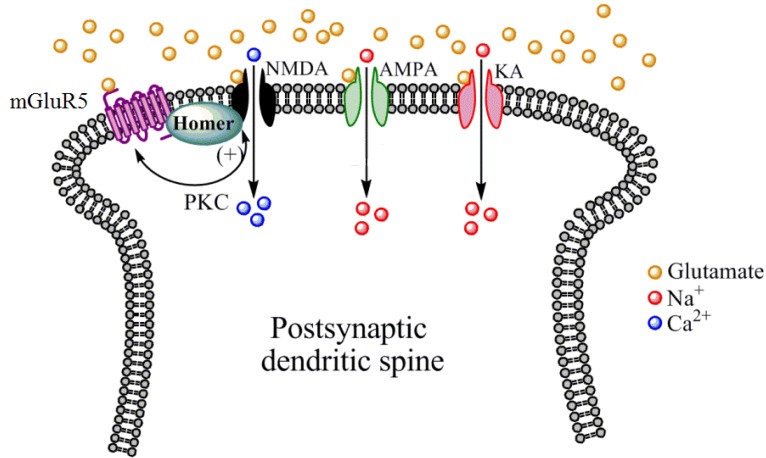
Mechanisms by which mGluR5 receptors indirectly modulate NMDA receptor function. Located primarily postsynaptically on dendritic spines, mGluR5 receptors are structurally linked to NMDA receptors by numerous scaffolding proteins including Homer proteins. Protein kinase C (PKC), which is activated by mGluR5 receptor stimulation, phosphorylates NMDA receptors to increase the cationic conductance of this receptor. PKC can also phosphorylate mGluR5 receptors to modulate their function. Other iGluR subtypes such as AMPA and KA do not appear to share this biochemical and structural linkage with mGluR5 receptors.

While the apparent lack of consistent effects of direct NMDA receptor modulators does not necessarily signify the end of the road for future addiction pharmacotherapy development targeting this receptor, it may be that more indirect approaches to altering NMDA receptor function are a more suitable approach. Along these lines, our laboratory has generated preclinical findings that mGluR5 PAMs, which indirectly enhance NMDA receptor function through biochemical and structural linkage between these two receptor subtypes (see Figure 3), facilitate extinction learning and reverse cognitive deficits in rodent models of addiction. For example, it has been shown that the mGluR5 PAM 3-cyano-N-(1,3-diphenyl-1H-pyrazol-5-yl) benzamide (CDPPB) facilitates the extinction of a cocaine conditioned place preference [98] as well as cocaine- and methamphetamine-seeking following intravenous self-administration [99,100]. CDPPB also reverses deficits in object recognition following methamphetamine self-administration [101]. mGluR5 PAMs are under currently development for reversing NMDA receptor hypofunctioning in schizophrenia [102], and thus may represent a novel approach for indirectly enhancing NMDA receptor function as a novel approach to enhancing cognition and reducing drug cue reactivity following CET. Likewise, mGluR5 NAMs which indirectly suppress NMDA receptor function are widely known to suppress drug intake and relapse-like behaviors in animal models [103,104,105,106].

Group I mGluRs represent just one of numerous alternative glutamatergic treatment approaches for SUDs [3,9,15]. The clinical efficacy of mGluR5 PAMs or NAMs in treating SUDs are currently known, since mGluR5 PAMs are still in preclinical development and no clinical trials to date have examined the ability of mGluR5 NAMs to reduce drug craving or intake. Regardless of the glutamatergic receptor target, it should be reiterated that no medication will be a universal treatment for all SUDs and behavioral addictions, but when properly used in combination with appropriate psychosocial, group, or cognitive-behavioral therapies, such compounds will hopefully improve treatment outcomes and reduce relapse rates.
